# The Increased Ratio of Blood CD56^bright^ NK to CD56^dim^ NK Is a Distinguishing Feature of Primary Sjögren's Syndrome

**DOI:** 10.1155/2020/7523914

**Published:** 2020-07-09

**Authors:** Bingxia Ming, Tong Wu, Shaozhe Cai, Peng Hu, Jungen Tang, Fang Zheng, Cong Ye, Lingli Dong

**Affiliations:** ^1^Department of Rheumatology and Immunology, Tongji Hospital, Tongji Medical College, Huazhong University of Science and Technology, Wuhan, Hubei, China; ^2^Department of Immunology, School of Basic Medicine, Tongji Medical College, Huazhong University of Science and Technology, Wuhan, China; ^3^Key Laboratory of Organ Transplantation, Ministry of Education; NHC Key Laboratory of Organ Transplantation; Key Laboratory of Organ Transplantation, Chinese Academy of Medical Sciences, Wuhan, Hubei, China

## Abstract

**Objective:**

The aim of this study was to characterize the subsets of circulating CD56^+^ NK cells in pSS patients and their potential value in the diagnosis and/or prediction of prognosis in patients with pSS.

**Methods:**

We included 52 pSS patients fulfilling the 2002 AECG criteria or 2012 ACR criteria and 20 age- and gender-matched healthy volunteers. The frequency and absolute number of NK cells and CD56 NK cell subsets in peripheral blood samples were detected by flow cytometry. Other laboratory parameters such as the IgG level and complement protein levels were extracted from the clinical system.

**Results:**

Both the frequency and the absolute number of peripheral blood NK cells were reduced in pSS patients compared to healthy controls. The proportion of CD56^bright^ NK cell subset was increased, and the proportion of CD56^dim^ NK cell subset was decreased among NK cells, resulting in the ratio of CD56^bright^ NK to CD56^dim^ NK which was significantly elevated in pSS patients. ROC analysis indicated that the AUC of CD56^bright^ NK/CD56^dim^ NK ratio was 0.838, and the best diagnostic cut-off point was 0.0487 for pSS patients. Furthermore, this CD56^bright^ NK/CD56^dim^ NK ratio was positively correlated with the IgG level and negatively correlated with the complement protein C3 and C4 levels. More importantly, the CD56^bright^/CD56^dim^ NK ratio was either slightly increased or not changed in other autoimmune diseases such as SLE and IgG4-related disease.

**Conclusion:**

Our findings suggest that the ratio of blood CD56^bright^ NK to CD56^dim^ NK might have a diagnostic value relatively specific for pSS.

## 1. Introduction

Primary Sjögren's syndrome (pSS) is a slowly progressed autoimmune disorder characterized by lymphocytic infiltration of exocrine glands and subsequent significant loss of secretory function with oral or eye dryness [1–3]. The diagnosis of pSS is based on the focal infiltration of mononuclear cells (mainly T and B cells) in glands and the presence of serum autoantibodies and hyperglobulinemia [4–8]. The above characteristics emphasize the role of abnormal adaptive immune responses in the pathogenesis of pSS. However, few studies have explored the role of innate immune indicators in the identification of pSS patients.

Natural killer (NK) cells are innate lymphoid cells that exhibit the capacity to secrete cytokines and possess natural cytotoxicity [9]. Although animal models of pSS have not directly implicated NK cells in disease pathogenesis, recent work implicates a regulatory role of NK cells in exocrine gland tissues and peripheral blood. For example, NK cells expressing NKp30 were proposed to interact with epithelial cells and subsequently mediate the enhancement of the inflammatory state in the salivary gland through secretion of interferon-*γ* (IFN-*γ*) [3]. Another study found that activated NK/NKT cells from pSS patients stimulated by interleukin-33 (IL-33) and IL-12/IL-23 could produce IFN-*γ* thereby perpetuating cellular damage [10]. In addition, increasing evidence has shown that NK cells play a critical role in both type I and type II IFN biologic functions resulting from their interaction with various dendritic cell (DC) subsets in pSS progression [11–14]. Taken together, these data suggest that NK cells play an important role in the pathogenesis of pSS.

NK cells are characterized conventionally by the expression of the CD56 surface marker [15, 16]. Based on the expression of CD56, human NK cells are divided into CD56^bright^ and CD56^dim^ subsets [17]. It is commonly recognized that CD56^bright^ NK cells account for about 10% of human peripheral blood NK cells mainly producing various cytokines and chemokines, whereas CD56^dim^ NK cells account for about 90% of human peripheral blood NK cells with higher cytotoxic [9, 18, 19]. CD56^bright^ and CD56^dim^ NK cells are successive stages in the development of NK cells. The circulating CD56^bright^ NK cells are generally considered to be the precursors of the CD56^dim^ NK cells [20]. A recent study has found CD56^high^ cells in the peripheral blood of newly diagnosed pSS patients were significantly reduced [21]. In contrast, another study published in 2013 showed that the proportion of circulating CD56^bright^ NK cells relative to the total NK cells was increased among pSS patients compared to controls [3]. The role of CD56^bright^ and CD56^dim^ NK subpopulations and their clinical significance in pSS is poorly understood. We hypothesize that a shifted balance between the CD56 NK cell subsets may reflect the immune status of pSS.

In this study, we analyzed the characteristics of peripheral blood CD56^bright^ NK cell subset and CD56^dim^ NK cell subset in patients with pSS and found that the ratio of CD56^bright^ NK to CD56^dim^ NK was elevated, and the area under the curve (AUC) of this ratio was 0.838 by receiver operator characteristic (ROC) curve analysis. Furthermore, this ratio was positively associated with serum IgG level and negatively associated with complement C3 and C4 levels, though not associated with EULAR Sjögren's Syndrome Disease Activity Index (ESSDAI) in pSS patients.

## 2. Materials and Methods

### 2.1. Patients and Healthy Volunteers

Independent identification and newly diagnosed patients with pSS, SLE, and IgG4-related disease (IgG4-RD) as well as healthy controls (HC) were included in this study. Samples of patients were obtained from the clinic of the Department of Rheumatology and Immunology, Tongji Hospital from November 2017 to November 2018. Patients with pSS (52), SLE [7], and IgG4-RD [22] fulfilled their respective classification criteria. Disease activity was scored by measuring the EULAR Sjögren's Syndrome Disease Activity Index (ESSDAI) in pSS. All subjects had neither a medical history of virus hepatitis, lymphoma, human immunodeficiency virus (HIV) infection, and diabetes nor a history of smoking, antiacetylcholine drugs, and radiation therapy for head and neck. Medical records were reviewed, and information regarding age, sex, and laboratory parameters was collected.

The approval of the ethical committee of Tongji Hospital, Tongji Medical College, Huazhong University of Science and Technology was obtained before the study. The ethical institutional review board (IRB) ID is TJ-C20151109. All subjects have provided written informed consent to participate in the study.

### 2.2. Cell Isolation and Flow Cytometry

Peripheral blood mononuclear cells (PBMCs) were isolated from heparinized blood samples from all patients and controls by Ficoll-Paque (GE Healthcare, Amersham, UK) density gradient centrifugation. Fluorochrome-conjugated antibodies specific for the following cell surface molecules were used: anti-human-CD3 (HIT3a)-PE and anti-human-CD56 (HCD56)-FITC (antibody reagents were purchased from Biolegend, UK). PBMCs were incubated with fluorochrome-conjugated antibodies diluted in PBS for cell surface staining at 4°C. Cells were subsequently washed and resuspended in PBS prior to analysis. Flow cytometry was carried out by using a Calibur flow cytometer (BD Biosciences, San Diego, CA), and data were analyzed using FlowJo 7.6 software (Tree Star, Inc., Ashland, OR). The lymphocyte population was identified by assessment of the size and granularity of cells using forward scatter (FSC) and side scatter (SSC). NK cell percentage expressed as a proportion of total gated lymphocytes. Among NK cells, CD56^bright^ and CD56^dim^ subsets were identified according to the level of CD56 expression. Gates were set by using isotype control antibodies.

### 2.3. Statistical Methods

GraphPad Prism 5.0 software was used for all statistical analyses. The Mann-Whitney *U* test was used to compare the independent groups. The ROC curve was used to analyze the diagnostic value of the ratio of CD56^bright^ NK to CD56^dim^ NK. Spearman's rank correlation test was applied to analyze the relationship between the two factors. *p* < 0.05 was considered statistically significant.

## 3. Results

### 3.1. Demographic Data of pSS Patients and Healthy Controls

The demographic characteristics and clinical features of pSS patients and healthy controls are described in Table 1. There was no significant difference in ages and sex between the groups.

### 3.2. Blood CD56 NK Cell Subset Levels in pSS Patients

**Figure 1:**
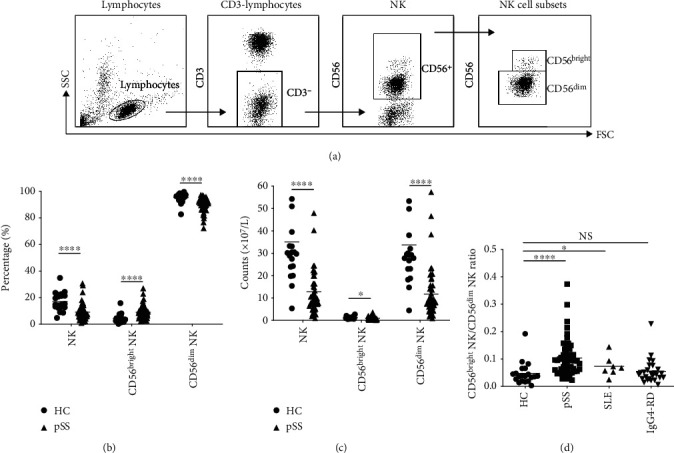
Comparison of percentage and counts of peripheral blood NK cells and CD56 NK cell subsets between pSS patients and HC. (a) The gating strategy is shown for the analysis of NK population. (b) The percentage of peripheral blood NK cells and their constituent CD56 subsets. (c) The absolute number of peripheral blood NK cells and their constituent CD56 NK cell subsets. (d) The ratio of CD56^bright^ NK/CD56^dim^ NK in autoimmune diseases. The horizontal line represents the mean. Definitions of abbreviations: HC = healthy controls; pSS = primary Sjögren's syndrome.

In accordance with previous literature [3], human NK cells defined as CD3^–^CD56^+^ lymphocytes in this study could be separated into CD56^bright^ and CD56^dim^ subsets in all pSS patients and HC (Figure 1(a)). The proportion of peripheral blood NK cells in total lymphocytes was reduced in pSS patients compared to healthy controls (Figure 1(b)). Among NK cells, the proportion of the CD56^bright^ NK cell subset was increased while the CD56^dim^ NK cell subset was decreased in patients of pSS (Figure 1(b)). The absolute number of NK cells and CD56 NK cell subsets was decreased, and the CD56^dim^ NK cell subset decreased more (Figure 1(c)). We have also detected the expression of CD107a and intracellular IFN-*γ*, which reflect the function of CD56^+^ NK cell subsets in pSS patients. CD56^bright^ NK cells and CD56^dim^ NK cells were presented with an increased percentage of CD107a^+^ cells and decreased IFN-*γ* expression (supplementary figure 1). The above results imply that the imbalance of CD56 NK cell subsets existed in pSS patients.

### 3.3. The CD56^bright^ NK to CD56^dim^ NK Ratio in the Identification of pSS Patients

We further calculated the ratio of CD56^bright^ NK cell subset to CD56^dim^ NK cell subset as described above and found that the ratio of CD56^bright^NK to CD56^dim^NK was significantly elevated in pSS patients than that in healthy controls (*p* < 0.0001, Figure 1(d)). Furthermore, the ratio of CD56^bright^ NK to CD56^dim^ NK was also analyzed in other autoimmune diseases, such as SLE and IgG4-RD. The ratio of CD56^bright^ NK to CD56^dim^ NK in peripheral blood of SLE patients was slightly increased (*p* = 0.041) and not different from those in healthy controls in patients with IgG4-RD (Figure 1(d)).

### 3.4. Sensitivity and Specificity of CD56^bright^ NK to CD56^dim^ NK Ratio in pSS Patients

**Figure 2:**
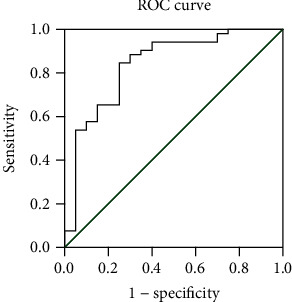
ROC curve of the ratio of CD56^bright^ NK to CD56^dim^ NK. The curve plots the relationship between sensitivity and 1 − specificity for different cut-off levels. When 0.0487 was used as the cut-off point for the diagnostic score of suspected pSS patients, the maximum value of the Youden index was achieved as 0.596.

ROC curve analysis indicated that the area under the curve (AUC) was 0.838, and the best diagnostic cut-off point of the ratio of CD56^bright^ NK to CD56^dim^ NK was 0.0487 for the patients with pSS (Figure 2). Using 0.0487 as the threshold value of the ratio, the sensitivity, specificity, and Youden index were 84.6%, 75%, and 0.596, respectively.

### 3.5. The Correlation of CD56^bright^ NK to CD56^dim^ NK Ratio with Disease Activity in pSS Patients

**Figure 3:**
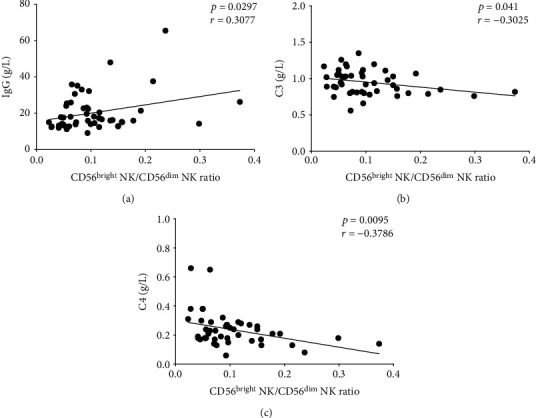
The correlation between CD56^bright^ NK/CD56^dim^ NK ratio and disease activity. (a) The correlation analysis between the CD56^bright^ NK/CD56^dim^ NK ratio and the serum IgG level or (b) complement C3 level or (c) complement C4 value in pSS patients.

We next analyzed the correlation of CD56^bright^ NK to CD56^dim^ NK ratio with disease activity in pSS patients. Spearman rank correlation did not identify a significant correlation of the ratio with the ESSDAI (supplementary figure 2). However, there was a significant correlation between the ratio and immunological features often associated with pSS, including IgG and complement C3 and C4 levels (Figures 3(a)–3(c)). These data suggest that the CD56^bright^ NK to CD56^dim^ NK ratio might be suitable for reflecting the immune status in pSS patients.

### 3.6. The Correlation of CD56^bright^ NK to CD56^dim^ NK Ratio with the Therapeutic Effect of pSS Patients

We have compared the CD56^bright^ NK, CD56^dim^ NK, and CD56^bright^ NK to CD56^dim^ NK ratio between before and after treatment in pSS patients. The drugs of treatment are mainly glucocorticoid and hydroxychloroquine. As shown in supplementary figure 1, ESSDAI and serum IgG level were decreased after treatment (supplementary figure 3D-E). The ratio and CD56^bright^ NK cell count were unchanged between before and after treatment, though the decreased CD56^dim^ NK cell count was upregulated after treatment in pSS patients (supplementary figure 3A-C).

## 4. Discussion

Increasing experimental evidence has shown a direct involvement of NK cells and CD56 NK cell subsets in some human immunopathologies such as psoriatic arthritis, SLE, multiple sclerosis, and Behcet's disease [19, 23–25]. However, as innate lymphoid cells, the role and clinical significance of CD56 NK cell subsets in pSS patients are poorly understood. In the present study, we compared the change of circulating CD56 NK cell subsets, firstly evaluated the value of CD56^bright^ NK to CD56^dim^ NK ratio in the diagnosis of pSS, and analyzed its association with clinical parameters.

As expected, we observed that the CD56^bright^ NK to CD56^dim^ NK ratio had the potential to diagnose pSS in autoimmune diseases. Using a cut-off value of 0.0487, we found that the sensitivity and specificity of the ratio were 84.6% and 75%, respectively. In order to further determine the significance of the CD56^bright^ NK to CD56^dim^ NK ratio in the identification of pSS patients, the changes of this ratio in other autoimmune diseases were analyzed. Accordingly, we found that the CD56^bright^ NK to CD56^dim^ NK ratio was also increased in SLE patients and unchanged in IgG4-RD. These distinct changes of the ratio in different autoimmune diseases may be attributed to their different pathological mechanisms. It has been reported that much similar pathogenesis have existed between patients with pSS and SLE [2, 22, 26], including the type I and type II IFN system in the innate immune phase, and autoantibodies such as anti-SSA/SSB and antinuclear antibody consistently appeared in both diseases, but not for IgG4-RD.

Due to the limited supply of fresh exocrine gland tissues, this study focused on the circulating NK cells in the peripheral blood of pSS patients. Both CD56^bright^ NK cells and CD56^dim^ NK cells were found to be depleted within the peripheral blood compartment of pSS patients compared to healthy controls, while the latter decreased more. We also found that among NK cells, the percentage of CD56^bright^ NK cells was increased and of CD56^dim^ NK cells was decreased, resulting from the greater loss of CD56^dim^ NK cell subset. Further analysis showed that the ratio of CD56^bright^ NK cells relative to the total NK population to CD56^dim^ NK cells was significantly elevated. Taken together, these results imply an imbalance of CD56 NK cell subsets in pSS, and this imbalance may reflect the immune status to some extent in pSS patients.

It is commonly recognized that serum IgG level, complement C3 and C4 consumption, and high ESSDAI are usually used to evaluate the disease activity in pSS patients. Goules et al. found that CD56^+^ NK cell incidence in the minor salivary glands of pSS patients was slightly increased, but not associated with the grade of inflammation [27]. Another previous study reported that CD56^+^ NK cell number in salivary glands was positively correlated to RF and serum C4 levels, and not correlated with the anti-SSA/SSB levels [28]. Recent research observed that the proportion of circulating CD56^bright^ NK cells was increased in pSS patients, though it was not associated with disease activity [3]. To confirm whether the CD56^bright^ NK to CD56^dim^ NK ratio could reflect the disease activity, we analyzed the correlation between this ratio and disease activity indexes. In agreement with the previously reported data about the CD56^+^ NK cells, we found that there is no significant correlation between the ratio and ESSDAI. Interestingly, we found this ratio was positively correlated with the serum IgG level and negatively associated with the complement C3 and C4 levels. Thus, our data are the in-depth study of the previous work about CD56 NK cell subsets in pSS.

In consistent with the previous report, our data showed that the proportion and absolute number of circulating CD3^−^CD56^+^ NK cells were significantly reduced in pSS patients versus controls [3, 29, 30]. The depletion of peripheral blood NK cells in pSS patients may be due to an increased homing of the cytotoxic cells to exocrine glands, which initiate and maintain tissue inflammation through the production of Th1 cytokines and cytotoxic mediators. In general, CD56^bright^ NK cells are mainly responsible for cytokine production, whereas CD56^dim^ NK cells are mostly cytolytic. However, recent studies have revealed CD56^dim^ NK cells to be a major source of cytokine production [31, 32]. Our data showed that circulating CD56^dim^ NK cell subsets exhibited a greater loss in pSS patients, maybe resulting in a more homing to the exocrine glands to promote the immune injury, which needs to be further confirmed.

There exist some limitations of this study. It is commonly recognized that CD56^+^ NK cell subsets in the salivary gland of pSS patients can be more suitable to reflect the situation in the gland. In this study, we mainly focused on the circulating CD56^+^ NK cell subsets. It is difficult to distinguish the CD56^bright^ and CD56^dim^ NK cell subsets by immunohistochemistry staining; also, the biopsy gland tissues of pSS patients are not enough to analyze with flow cytometry. In addition, the number of SLE patients was relatively small. We only included newly diagnosed SLE patients as controls, which limited the number of included patients.

In summary, our results suggest that the ratio of peripheral blood CD56^bright^ NK to CD56^dim^ NK has the potential to identify the pSS patients and was associated with serum IgG levels and complement C3 and C4 consumption. Further studies are required to better understand the crucial role of CD56 NK cell subsets in the pathogenesis of pSS in disease-affected tissues.

## Figures and Tables

**Table 1:** Overview of individuals at baseline used in this study.

	pSS	HC
Characteristics		
Number	52	20
Age in years (mean ± SD)	47.7 ± 11.3	46.8 ± 12.0
Range	20-68	24-77
Female : male	49 : 3	19 : 1
Clinical features		
ANA+ (%)	44 (84.6)	0 (0)
Ro/SSA+ (%)	44 (84.6)	NA
La/SSB+ (%)	23 (44.2)	NA
ESR, high levels (>20 mm/h) (%)	25 (48.1)	NA
CRP, high levels (>5 mg/l) (%)	7 (13.5)	NA
IgG, elevated levels (>16 g/l) (%)	26 (50)	NA
Focus score ≥ 1 (%)	42 (87.5)^a^	NA
ESSDAI range (median)	0-15 (4)	NA

Categorical data are expressed as the absolute frequency, with percentages in parenthesis. ANA: antinuclear antibodies; Ro/SSA+: antibodies against Ro/Sjögren's syndrome A antigen; La/SSB+: antibodies against La/Sjögren's syndrome B antigen; ESR: erythrocyte sedimentation rate; CRP: C-reactive protein; focus score indicates the number of inflammatory foci containing more than 50 mononuclear cells per 4 mm^2^ biopsy tissue. ESSDAI: EULAR Sjögren's Syndrome Disease Activity Index; NA: not applicable. ^a^*n* = 48.

## Data Availability

The data that support the findings of this study are available. If it is necessary, we will provide it at all.

## Supplementary Materials

Supplementary materials Figure 1: comparison of function of peripheral NK cells and CD56 NK cell subsets between pSS patients and HC. (A) The percentage of CD107a^+^ cells in NK cells or CD56 NK cell subsets. Cells were collected from HCs (*n* = 8) and pSS patients (*n* = 15). (B) The percentage of intracellular expression of IFN-*γ* gated from NK cells or CD56 NK cell subsets. Cells were collected from HCs (*n* = 10) and pSS patients (*n* = 18). HC = healthy control; pSS = primary Sjögren's syndrome. Figure 2: the correlation between CD56^bright^ NK/CD56^dim^ NK ratio and ESSDAI in pSS patients. Definitions of abbreviations: ESSDAI: EULAR Sjögren's Syndrome Disease Activity Index. Figure 3: the change of peripheral NK cells and CD56 NK cell subsets in pSS patients (*n* = 12) before and after treatment. (A) The absolute number of peripheral CD56^dim^ NK cell subsets, (B) the absolute number of peripheral CD56^bright^ NK cell subsets, (C) the ratio of CD56^bright^ NK/CD56^dim^ NK, (D) ESSDAI, and (E) Serum serum IgG level before and after treatment.
