# Supplementary material for: The Increased Ratio of Blood CD56bright NK to CD56dim NK Is a Distinguishing Feature of Primary Sjögren's Syndrome
Source: J Immunol Res. 2020 Jul 9;2020:7523914. doi: 10.1155/2020/7523914 (PMC7368214; doi:10.1155/2020/7523914)
Supplement: Supplementary materials — Figure 1: comparison of function of peripheral NK cells and CD56 NK cell subsets between pSS patients and HC. (A) The percentage of CD107a+ cells in NK cells or CD56 NK cell subsets. Cells were collected from HCs (n = 8) and pSS patients (n = 15). (B) The percentage of intracellular expression of IFN-γ gated from NK cells or CD56 NK cell subsets. Cells were collected from HCs (n = 10) and pSS patients (n = 18). HC = healthy control; pSS = primary Sjögren's syndrome. Figure 2: the correlation between CD56bright NK/CD56dim NK ratio and ESSDAI in pSS patients. Definitions of abbreviations: ESSDAI: EULAR Sjögren's Syndrome Disease Activity Index. Figure 3: the change of peripheral NK cells and CD56 NK cell subsets in pSS patients (n = 12) before and after treatment. (A) The absolute number of peripheral CD56dim NK cell subsets, (B) the absolute number of peripheral CD56bright NK cell subsets, (C) the ratio of CD56bright NK/CD56dim NK, (D) ESSDAI, and (E) Serum serum IgG level before and after treatment. [file 7523914.f1.zip › mat.7523914.v2.doc]

**Supplementary figure 1**

**Comparison of function of peripheral NK cells and CD56 NK cell subsets between pSS patients and HC.** (A) The percentage of CD107a^+^ cells in NK cells or CD56 NK cell subsets. Cells were collected from HCs (n=8) and pSS patients (n=15). (B) The percentage of intracellular expression of IFN-γ gated from NK cells or CD56 NK cell subsets. Cells were collected from HCs (n=10) and pSS patients (n=18). HC=healthy control, pSS=primary Sjögren’s syndrome.

**Supplementary figure 2**

**The correlation between CD56^bright^ NK/CD56^dim^ NK ratio and ESSDAI in pSS patients.** Definitions of abbreviations: ESSDAI, EULAR Sjögren’s Syndrome Disease Activity Index.

**Supplementary figure 3**

**The change of peripheral NK cells and CD56 NK cell subsets in pSS patients (n=12) before and after treatment.** (A) The absolute number of peripheral CD56^dim^ NK cell subsets, (B) the absolute number of peripheral CD56^bright^ NK cell subsets, (C) the ratio of CD56^bright^ NK/CD56^dim^ NK, (D) ESSDAI and (E) Serum IgG level before and after treatment.
